# Opening Side of Unilateral Open-Door Laminoplasty Does Not Impact Improvement in Arm Pain or Space Available for the Spinal Cord

**DOI:** 10.3390/jcm13113345

**Published:** 2024-06-06

**Authors:** Robert K. Merrill, Tejas Subramanian, Tomoyuki Asada, Sumedha Singh, Amy Lu, Max Korsun, Omri Maayan, Izzet Akosman, James Dowdell, Russel C. Huang, Sravisht Iyer, Todd J. Albert, Francis Lovecchio, Han Jo Kim

**Affiliations:** 1 Department of Spine Surgery, Hospital for Special Surgery, New York, NY 10021, USA; 2 Weill Cornell Medicine, New York, NY 10021, USA

**Keywords:** laminoplasty, open door, laterality, patient outcomes, arm pain

## Abstract

**Background/Objectives**: There exists limited data guiding open-door laminoplasty. The objective of this study is to determine if open-door laminoplasty affects radiographic decompression or arm pain outcomes. **Methods**: Adult patients who underwent unilateral open-door laminoplasty cervical myelopathy were included. The side opened was dependent on surgeon discretion. We recorded preoperative side of symptoms, side of radiographic compression, arm pain scores, and canal diameter. Patients with open-side ipsilateral or contralateral to dominant symptoms or compression were compared to determine any effect on arm pain outcomes or spinal canal diameter. If the symptoms were equal bilaterally, patients were neutral. **Results**: A total of 167 patients were included, with an average age of 64 ± 11 years and average follow-up time of 64.5 ± 72 weeks. The average preoperative arm pain visual analog score (VAS) was 2.13 ± 2.86, and the average arm VAS after 6 months was 1.52 ± 2.68. For dominant symptoms, the ipsilateral, contralateral, and neutral groups had a significant improvement in arm VAS at >6 months postoperatively. For dominant compression, the ipsilateral and contralateral groups had a significant improvement in both arm VASs and canal diameter at >6 months postoperatively. No differences were seen between groups for either. We observed a significant correlation between size of plate and change in canal diameter; however, no differences were noted for arm pain. **Conclusions**: Laminoplasty may be effective in addressing radicular arm pain by increasing the spinal canal’s diameter and space available for the cord. The laterality of open-door laminoplasty did not affect arm pain improvement or canal expansion.

## 1. Introduction

Initially described in 1973 by Oyama et al., laminoplasty has become a popular surgical treatment option for degenerative cervical myelopathy [1,2]. The original technique devised by Oyama involved z-plasty cuts of each lamina, which were then lifted and fixed with suture. The purpose of this technique was to address certain issues related to laminectomy, such as post-laminectomy kyphosis and instability [3]. This technique went through several iterations, including a procedure described by Tsuji, where the lamina was cut bilaterally at the lamina–facet junction and simply allowed to float on the spinal cord without any fixation [4]. Hirabayashi et al. then described open-door laminoplasty, where one side of the lamina at the lamina–facet junction was completely cut, and the other side was only cut unicortically. The lamina would then be opened like a door on a hinge [5]. The next major iteration was described by Kurokawa, where the spinous processes were split down the middle and hinged open bilaterally, commonly referred to as the French-door technique [6]. Today, French door and open door are the two most commonly used laminoplasty techniques, with no clear superiority demonstrated by either [7,8,9]. While traditionally indicated for degenerative cervical myelopathy, laminoplasty has shown benefits in the treatment of radicular arm pain, either alone or in conjunction with foraminotomy procedures [10,11].

There are no clear guidelines to direct the decision of which side to open during open-door laminoplasty, and it is generally left to surgeon discretion and preference. The first study published on this subject was by Tang et al. in 2020, and was performed only on patients with cervical myelopathy secondary to the ossification of the posterior longitudinal ligament (OPLL) [12]. The results suggest that, when the OPLL is not midline, opening the side contralateral to the OPLL leads to improved myelopathy outcomes. In a more recent study by Kang et al., open-door laminoplasty had no impacts on myelopathy, radiculopathy, or radiographic outcomes, regardless of the preoperative symptom side or side of radiographic compression [13]. Whether the opening side of unilateral open-door laminoplasty affects outcomes therefore remains unclear.

The objective of our study was to investigate if open-door laminoplasty ipsilateral or contralateral on either the side of radiologic compression or the side of arm symptoms played any role in the improvement of arm symptoms or the expansion of the spinal canal postoperatively. These results may provide surgeons with objective criteria for determining which side to open during laminoplasty. Secondarily, we determined if the size of plate used affected canal expansion, and if canal expansion improved symptoms.

## 2. Materials and Methods

### 2.1. Patient Sample

This study was conducted after Institutional Review Board approval and informed consent was obtained for all patients. We performed a multi-surgeon retrospective review of all patients who underwent a C3–7 unilateral open-door laminoplasty for a primary indication of cervical spondylotic myelopathy with or without radicular symptoms in 2017–2022. Each participating surgeon was considered at least an experienced specialist with at least 5 years’ experience according to Tang et al. [14]. The standard procedure performed at our institution is to perform a dome laminectomy of the distal portion of C3 and proximal portion of C7, and laminoplasty of C4, C5, and C6. The open-door side is chosen based on each surgeon’s preference. The main determinant of the opening side corresponds to which side of the table the surgeon is most comfortable standing on during surgery. If the surgeon stands on the patient’s left side, the left side is opened during laminoplasty. Only adult patients ≥18 years of age were included in the study. Patients who had a concomitant foraminotomy performed at any level on either the left or right side in addition to laminoplasty were excluded from the study. Patients who underwent fewer levels than C3–7, or who had hybrid procedures, such as two level laminoplasties with a single-level laminectomy and/or fusion, were also excluded. Patient demographic and operative data, including age, gender, body mass index (BMI), Charlson comorbidity index (CCI), and operative time, were all extracted.

### 2.2. Symptom Side, Compression Side, and Space Available for the Cord

The dominant-symptom side was determined from the most recent preoperative clinic note as the patient’s description of symptoms being either left-sided, right-sided, or equal bilaterally.

**Figure 1:**
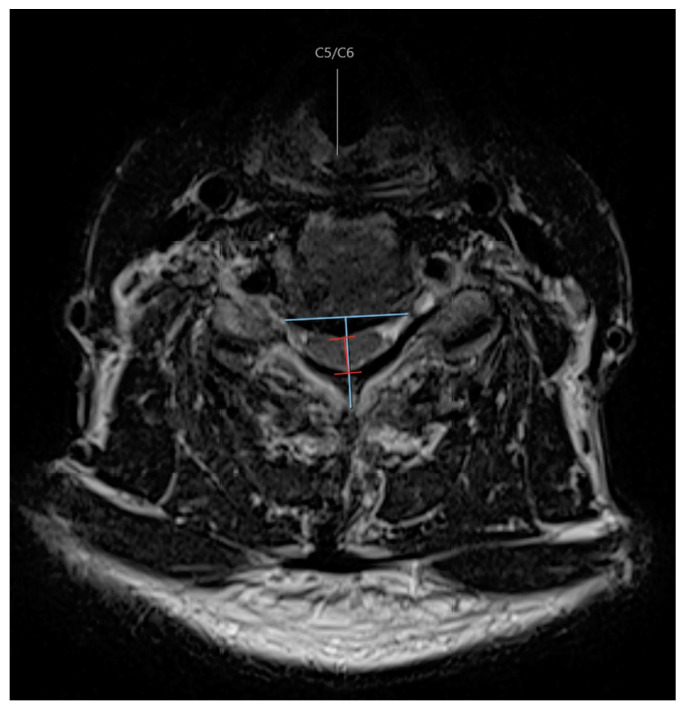
Axial T2-weighted MRI demonstrating radiographic measurements to determine the side of compression and canal diameter.

The radiographic compression side was determined by a line estimated to be perpendicular to the midline of the posterior aspect of the vertebral body/disc of the level in question, and if the apex of maximal compression was located to the right or left of that line (Figure 1). This was measured at each level on axial T2-weighted MRI images by two independent reviewers with a specialty in spine surgery. If there was a discrepancy about the side of compression at any level for any patient, then a third reviewer was used. The third reviewer used the same criteria, and the majority of the three reviewers was used to designate the compression side. To assign each patient with an overall classification of the side of compression, if most levels were compressed on one side, this was considered the dominant side of compression. If there was an equal number of levels compressed on the right and left sides, then the most stenotic level dictated the overall compression side. This allowed us to create a group of patients that had the opening side of laminoplasty on either the ipsilateral or contralateral side to dominant radiographic compression.

Space available for the spinal cord (SAC) was measured at each level, where maximal compression was noted on T2-weighted axial images. The SAC was measured as the diameter of the spinal canal on midline axial images considered the maximal distance from the source of compression to the posterior elements. The SAC was taken as the average between two independent reviewers.

### 2.3. Outcome Measures

Visual analog scale (VAS) arm and neck pain scores were collected from the patient chart at the most recent preoperative visit and at two weeks, six weeks, three months, six months, one year, and two years. Outcome scores were coalesced for each patient into less than six months, and greater than or equal to six-month scores. Additionally, postoperative complications, including medical complications, infections, residual symptoms, and any resulting reoperations, were extracted from patient charts. The number of patients included at each follow up can be found in Supplemental Table S1.

### 2.4. Statistical Analysis

All statistical analysis was performed with R (R foundation for statistical computing, Vienna, Austria, Version 4.2.1 accessed on 1 May 2023).

The Shapiro–Wilk normality test was performed to determine the distribution of each variable. For univariate analysis, a two-tailed *t*-test and Wilcoxon rank sum test were used to compare normal and non-normal continuous variables, respectively, and a chi-squared test was used to compare categorical variables between groups.

A Cohen’s kappa was used to calculate inter-rater reliability for determining compression side on the radiographs.

To determine if the opening side influenced arm pain outcomes, we divided the patients into groups based on whether the laminoplasty side was opened ipsilateral (I) or contralateral (C) to the side of symptoms. If the symptoms were equal bilaterally, this was considered a neutral (N) group. A two-way analysis of variance (ANOVA) with repeated measures was performed using VASs as the dependent variable and timepoint and patient group (I, C, or N) as the independent variables.

We performed another two-way ANOVA with repeated measures, but grouped patients based on the opening side ipsilateral (I) or contralateral (C) to the side of preoperative radiographic compression. There was no neutral group. We repeated this process using VASs as the dependent variable, but also used SAC at each level as the dependent variable to determine if openings ipsilateral or contralateral to the side of compression affected the SAC.

Lastly, we performed a Pearson’s correlation to determine if there was any correlation between plate size and canal expansion from pre- to postoperative stages, and if there was a correlation between canal expansion and change in VASs from pre- to postoperative stages.

Statistical significance was determined at a *p*-value of 0.05.

## 3. Results

### 3.1. Patient Sample

A total of 167 patients were included in the study with an average age of 64 years (range: 28–91 years old) and average follow-up time of 64.5 weeks. Table 1 summarizes some of the operative and demographic characteristics of the cohort. There were no statistically significant differences between opening sides with respect to patient age, American Society of Anesthesiology class, Charlson comorbidity index, or BMI. Average follow-up time was 456 ± 486 days. For patients who had less than a 6-month follow up, the average follow-up time was 44 ± 15 days. For those who had greater than a 6-month follow up, the average follow-up time was 555 ± 492.

### 3.2. Does the Opening Side Relative to Symptom Side Affect the Improvement in Arm Symptoms?

**Figure 2:**
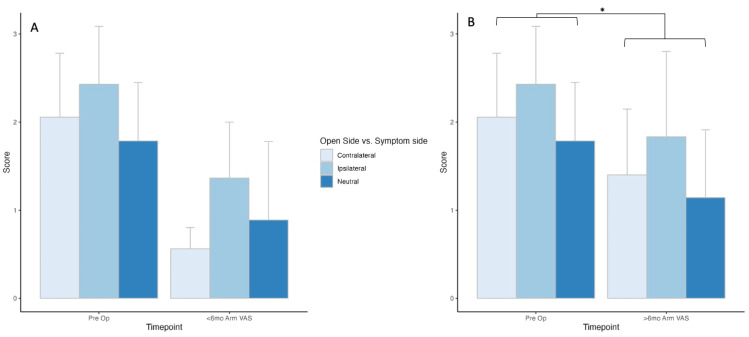
Early and late-timepoint VAS arm PROM outcomes comparing open-side versus symptom-side patients. * Denotes statistical significance of *p* < 0.05.

Comparing the opening side relative to the symptom side presented 18 (contralateral), 21 (ipsilateral), and 14 (neutral) patients with outcome scores for the preoperative timepoint, and 16 (contralateral), 11 (ipsilateral), and 9 (neutral) patients at the less-than-6-month postoperative timepoint. There was no statistically significant difference in outcomes between timepoints (*p* = 0.33) or among groups (*p* = 0.57), and no interaction between timepoint and groups (*p* = 0.30) (Figure 2A).

There were 10 (contralateral), 12 (ipsilateral), and 7 (neutral) patients with outcome scores for the greater-than-6-month timepoint. There was no statistically significant difference in outcomes among groups (*p* = 0.42) and no interaction between timepoint and groups (*p* = 0.59), but there was a statistically significant improvement in outcome scores from pre- and postoperative timepoints (*p* = 0.02, Figure 2B).

### 3.3. Does the Opening Side Relative to the Compression Side Affect the Improvement in Arm Symptoms?

The inter-rater reliability of determining the preoperative compression side was 75%.

**Figure 3:**
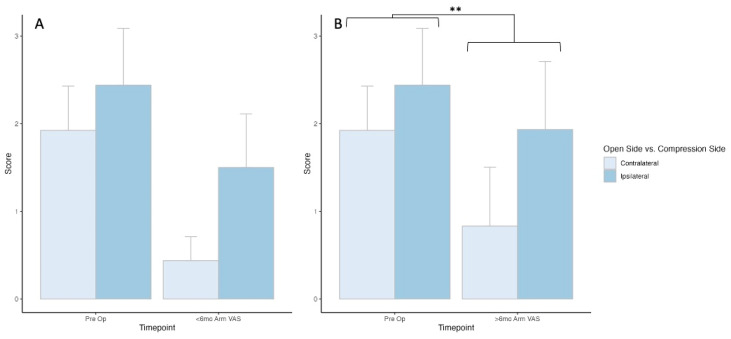
Early and late-timepoint VAS arm PROM outcomes comparing open-side versus compression-side patients. ** Denotes statistical significance of *p* < 0.05.

Comparing the opening side relative to the symptom side presented 26 (contralateral) and 25 (ipsilateral) patients with outcome scores at the preoperative timepoint, and 16 patients at the less-than-6-month postoperative timepoint for both the ipsilateral and contralateral groups. There was no statistically significant difference in outcome scores between timepoints (*p* = 1) or between groups (*p* = 0.21), and no interaction between time and group (*p* = 0.38, Figure 3A).

For the greater-than-6-month timepoint, there were 12 (contralateral) and 15 (ipsilateral) patients with outcome scores. There was no statistically significant difference in outcome scores between groups, and no interaction between groups and timepoints, but there was a statistically significant improvement in outcome scores between pre- and postoperative greater-than-6-month timepoints (*p* = 0.009) (Figure 3B).

### 3.4. Does the Opening Side Relative to the Side of Dominant Compression Affect the Expansion of the Spinal Canal?

For patients who had a postoperative MRI completed, the average time for the MRI performed was 1.4 years.

**Figure 4:**
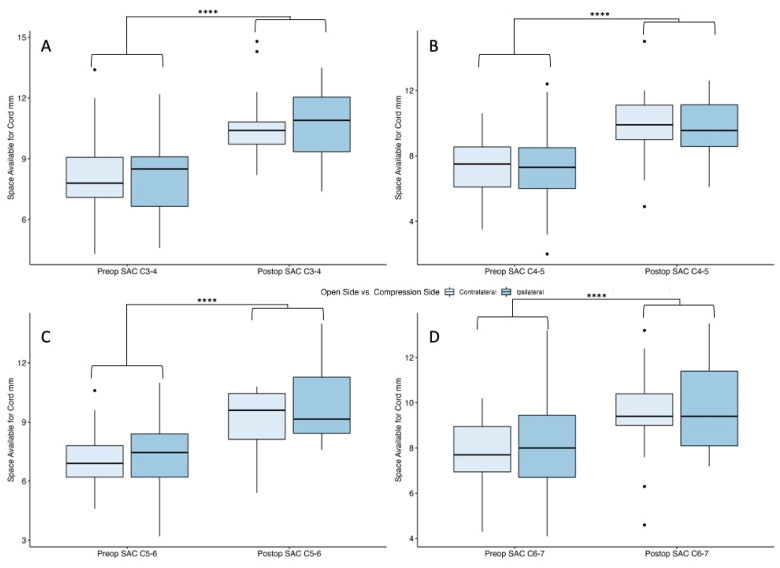
Preoperative versus postoperative SAC comparing open-side versus compression-side patients at levels (**A**) C3–C4, (**B**) C4–C5, (**C**) C5–C6, and (**D**) C6–C7. **** Denotes statistical significance of *p* < 0.05.

The C3–4 level had 58 (contralateral) and 59 (ipsilateral) patients in the preoperative timepoint and 20 (contralateral) and 19 (ipsilateral) patients in the postoperative timepoint. There was no statistically significant difference between groups (*p* = 0.88) and no interaction effect (*p* = 0.46), but there was a statistically significant increase in canal diameter from pre- to postoperative stages (*p* < 0.0001) (Figure 4A).

The C4–5 level had 59 (contralateral) and 67 (ipsilateral) patients in the preoperative timepoint and 17 (contralateral) and 24 (ipsilateral) patients in the postoperative timepoint. There was no statistically significant difference between groups (*p* = 0.77) and no interaction effect (*p* = 0.99), but there was a statistically significant increase in canal diameter from pre- to postoperative stages (*p* < 0.0001) (Figure 4B).

The C5–6 level had 57 (contralateral) and 62 (ipsilateral) patients in the preoperative timepoint and 12 (contralateral) and 26 (ipsilateral) patients in the postoperative timepoint. There was no statistically significant difference between groups (*p* = 0.42) and no interaction effect (*p* = 0.31), but there was a statistically significant increase in canal diameter from pre- to postoperative stages (*p* < 0.0001) (Figure 4C).

The C6–7 level had 51 (contralateral) and 50 (ipsilateral) patients in the preoperative timepoint and 17 (contralateral) and 17 (ipsilateral) patients in the postoperative timepoint. There was no statistically significant difference between groups (*p* = 0.61) and no interaction effect (*p* = 0.77), but there was a statistically significant increase in canal diameter from pre- to postoperative stages (*p* < 0.0001) (Figure 4D).

### 3.5. Does Plate Size Correlate with Canal Expansion, and Does Canal Expansion Correlate with the Improvement in Arm Symptoms?

One-hundred-and-fifty-seven patients had information available for plate size. There was a statistically significant positive correlation between plate size and canal expansion at the C3–4 (*p* = 0.005), C4–5 (*p* = 0.01), C5–6 (*p* = 0.006), and C6–7 (*p* = 0.01) levels. Table 2 demonstrates the mean change in canal diameter based on plate size.

There was no statistically significant correlation between canal expansion at any level and improvement in arm symptoms at either the <6 or >6-month timepoints.

### 3.6. Complications

There was a total of 17 (10.2%) complications. Five patients experienced wound infections. Of these, only one required operative irrigation and debridement. The remaining four resolved with oral antibiotics. Four patients required a return to the OR necessitating anterior cervical decompression and fusion. Three of these patients had residual myelopathy with single-level central stenosis, and one patient had a herniated disc causing radiculopathy at the level below the laminoplasty. The average time for these four patients returning to the OR was 16 months after the index operation. Four patients developed medical complications requiring medical intervention, three developed either post-laminoplasty kyphosis or distal junctional issues, and only one patient developed a C5 palsy that resolved completely by 6 weeks.

There was no statistically significant difference in the incidence of complications if the opening side was contralateral or ipsilateral to either symptoms (*p* = 0.88) or compression (*p* = 0.16).

## 4. Discussion

Laminoplasty is a technique commonly used to treat cervical myelopathy for degenerative spondylotic reasons or secondary to OPLL. Though, studies suggest that radicular pain secondary to foraminal stenosis may also improve after laminoplasty procedures [10,11]. The present study sought to clarify if an opening on a particular side of the laminoplasty leads to an improvement in arm symptoms depending on the preoperative side of symptoms or compression. We observed an improvement in mean arm VAS from pre- to greater-than-6-months postoperatively, with no effect of the opening side on arm symptom improvement. We secondarily determined if the plate size or if the opening side relative to the side of compression affected spinal canal expansion. We observed an increase in canal expansion from pre- to postoperative stages at all laminoplasty levels. There was a positive correlation between plate size and canal expansion, and the opening side did not affect canal expansion.

The first study to Investigate this topic to our knowledge was by Tang et al. in 2020 [12]. The authors investigated only patients who had myelopathy secondary to OPLL, but excluded any patient who had a diagnosis of radiculopathy. Outcomes were evaluated by the Japanese Orthopedics Association (JOA) score and spinal canal enlargement rate at a two-year follow up. The authors found that postoperative JOA scores (13.0 ± 1.4 vs. 12.1 ± 1.1) and the JOA recovery rate (49.6% ± 11.5 vs. 39.6% ± 8.8) were both higher in the group with the open side contralateral to the OPLL. Their results were supported by an increase in the cross-sectional spinal canal area (0.19 ± 0.05 cm^2^ vs. 0.09 ± 0.05 cm^2^) in the group where the open side was contralateral to the OPLL. This study differs from ours in that it exclusively investigated patients with OPLL and excluded patients with radiculopathy. The main goal of the study was to determine improvements in myelopathy and spinal canal expansion. The authors did not record the dominant symptom side and also did not specify how they chose which side to open. Our results cannot directly compare with Tang et al.’s analysis as we also included patients without OPLL. We found a statistically significant increase in canal diameter after laminoplasty at each level, but this did not change, depending on whether the open side was ipsilateral or contralateral to the dominant region of compression. Using a larger plate correlated with a larger expansion of the canal, but this did not affect outcomes for arm symptoms. Future studies should clarify the relationship between the magnitude of canal expansion and magnitude of clinical improvement, since larger plates may be more beneficial if a relationship is found.

A second radiographic study by Hua et al. corroborates the results by Tang and colleagues [15]. These authors also exclusively studied patients with myelopathy secondary to OPLL. They divided patients into “good” and “poor” groups based on a JOA recovery rate ≥ 50% or <50%, respectively. The authors found both a canal occupation ratio >60% and an opening on the ipsilateral side to compression were risk factors for having a poor outcome. It is important to understand that our study and Tang et al.’s and Hua et al.’s studies are not evaluating the same pathology or outcome. We investigated improvements in radicular pain and canal expansion in any patient undergoing laminoplasty. Therefore, the compression in our patients may not be from OPLL. It is important to consider these parameters when deciding which laminoplasty side to open. While our results suggest that canal expansion does not depend on the opening side, it is important to still consider the results of previous literature showing openings on the contralateral side of compression may produce larger canal expansion and improvements in myelopathy when compression is secondary to OPLL [12,15,16].

The most recent study on the topic was published by Kang et al., in which the authors investigated all patients who underwent laminoplasty for degenerative cervical myelopathy/radiculopathy with a two-year follow up [13]. Patients were divided into groups based on dominant compressive side, dominant myelopathy side, and dominant radiculopathy side. All patients had the laminoplasty performed on the right side and no differences were seen in any outcome at final follow up, suggesting that opening side relative to compression or symptoms does not matter. Our results support these findings, in that we saw an improvement in arm symptoms at the final follow up, independent of opening ipsilateral or contralateral to the preoperative symptom side. An important note is that Kang et al. performed foraminotomy at any level with foraminal stenosis and radiculopathy. This may confound the effects of the opening side of the laminoplasty relative to symptoms. We excluded patients who had a concomitant foraminotomy, and therefore the improvements in arm symptoms we observed were exclusively from decompression due to the laminoplasty itself.

Laminoplasty likely plays a role in the improvement of radicular pain through the decompression of nerve root takeoff. This occurs on both the open-door side as well as the hinge side. Plate size and canal expansion likely do not correlate with the improvement in arm symptoms because, once the lamina is elevated, the entirety of the nerve root takeoff is decompressed, and no further decompression would be provided by a large plate or larger central canal expansion.

In summary, our results provide some evidence to suggest that laminoplasty alone may sufficiently treat radiculopathy. Our patients had quite low arm VASs (2.13 ± 2.86) preoperatively, likely a result of selection bias for myelopathy being the primary indication for surgery. Though we cannot strongly conclude that laminoplasty treats radiculopathy, our results suggest that, in patients with mild arm symptoms secondary to radiculopathy, open-door laminoplasty alone, regardless of opening laterality, can result in a resolution of radiculopathy without the need for foraminotomy. Further studies should investigate the effects of laminoplasty alone on patients with severe radiculopathy. The preoperative side of dominant radiculopathy should not influence the choice of which side to open. The openside during laminoplasty should be up to the surgeon’s level of comfort and preference. Lastly, while we demonstrated a correlation between large-size plates and increase in canal expansion, there was no correlation between canal expansion and improvement in arm symptoms. This suggests that plates should be chosen based on patient anatomy and goodness of fit, rather than on trying to achieve the largest canal diameter. Future studies, though, are necessary to investigate the relationship between canal expansion and improvement in outcomes.

Our study is not without limitations. This was a retrospective multi-surgeon review, and therefore surgical techniques may vary slightly between surgeons. Side of laminoplasty opening was based primarily on surgeon preference and not objective criteria. While each surgeon performs a unilateral open-door laminoplasty technique, slight technical variations in some aspects of the procedure among surgeons may contribute to outcome differences, and we could not capture this. This allowed us to include both right- and left-side openings, which is a strength of the study, but a limitation in that it adds technical heterogeneity. Additionally, as a retrospective review, interpretations of dominant symptom side from surgeon clinic notes may be unreliable. However, given the standardized documenting protocols at our institution, we believe reliability issues have been mitigated. As previously mentioned, there was a selection bias for patients with a primary diagnosis of myelopathy, and therefore, while we aimed to report on improvements in radicular arm pain, the patients chosen for the study primarily had myelopathy. Additionally, the correlation of the arm pain to the compression level was not evaluated. We also did not have a granular breakdown of diagnosis based on degenerative conditions versus other pathologies, such as OPLL, adding to the heterogeneity of the patient sample. Lastly, while we had a cohort of 167 patients, there were much fewer patients with outcome data at all timepoints, which were necessary to conduct paired statistical testing. This may significantly reduce the power of our analysis and introduce another potential selection bias. It also prevented us from providing details on outcomes at more specific timepoints. Our conclusions are thus conditioned to our statistical limitations.

## 5. Conclusions

Unilateral open-door laminoplasty is a common procedure used to treat cervical myelopathy. The technique is also effective at reducing arm symptoms and increasing spinal canal diameter. Both the improvement in arm symptoms and expansion of the spinal canal do not depend on which side the laminoplasty is performed relative to preoperative symptoms or the compression side. Lastly, using larger laminoplasty plates leads to a large expansion of the spinal canal, but this does not influence the improvement in arm symptoms.

## Figures and Tables

**Table 1:** Characteristics of the patient cohort.

Variable	N = 167
Age (mean (SD))	64.49 (11.47)
Gender (%)	
Male	105 (62.9)
Female	62 (37.1)
BMI (mean (SD))	28.68 (5.83)
Charlson Comorbid Index (mean (SD))	0.64 (1.22)
Operative Time in Minutes (mean (SD))	157.47 (60.57)
Open side (%)	
Right	103 (61.7)
Left	64 (38.3)
Dominant Side of Symptoms (%)	
Bilateral equally	57 (34.1)
Left	56 (33.5)
Right	54 (32.3)
Dominant side of compression (%)	
Right	80 (51.9)
Left	87 (48.1)
Open Side Relative to Symptom Side (%)	
Contralateral	42 (25.1)
Ipsilateral	68 (40.7)
Neutral	57 (34.1)
Open side relative to compression side (%)	
Ipsilateral	85 (55.2)
Contralateral	82 (44.8)
Preoperative arm VAS (mean (SD))	2.13 (2.86)
Less-than-six-month arm VAS (mean (SD))	0.89 (1.85)
Greater-than-six-month arm VAS (mean (SD))	1.52 (2.68)

**Table 2:** Average canal expansion depending on size of plate used at each level.

Variable	8 mm Plate	10 mm Plate	12 mm Plate
n	41	85	31
Canal Expansion at C3–4 in mm (mean (SD))	1.11 (2.35)	2.24 (1.67)	5.49 (4.14)
Canal Expansion at C4–5 in mm (mean (SD))	2.22 (1.93)	2.15 (2.02)	5.19 (3.30)
Canal Expansion at C5–6 in mm (mean (SD))	1.70 (0.90)	2.81 (1.59)	3.96 (4.39)
Canal Expansion at C6–7 in mm (mean (SD))	1.36 (1.57)	1.45 (2.02)	3.88 (5.00)

## Data Availability

The data presented in this study are not available to the public but would be available upon reasonable request.

## Supplementary Materials

The following supporting information can be downloaded at: https://www.mdpi.com/article/10.3390/jcm13113345/s1; Table S1. Number of patients included for each analysis.
