# Supplementary material for: Opening Side of Unilateral Open-Door Laminoplasty Does Not Impact Improvement in Arm Pain or Space Available for the Spinal Cord
Source: J Clin Med. 2024 Jun 6;13(11):3345. doi: 10.3390/jcm13113345 (PMC11172582; doi:10.3390/jcm13113345)
Supplement: Supplementary file 1 [file jcm-13-03345-s001.zip › jcm-2994193-supplementary.pdf]

Supplemental Table S1. Number of patients included for each analysis.

<table>
<tr><th>Question</th><th>Preoperative</th><th><6-month</th><th>≥6-month</th></tr>
<tr><td>Does Opening Side Relative to Symptom Side Affect Improvement in Arm symptoms?</td><td>18 (contralateral), 21 (ipsilateral), and 14 (neutral)</td><td>16 (contralateral), 11 (ipsilateral), and 9 (neutral)</td><td>10 (contralateral), 12 (ipsilateral), and 7 (neutral)</td></tr>
<tr><td>Does Opening Side Relative to Compression Side Affect Improvement in Arm symptoms?</td><td>26 (contralateral) and 25 (ipsilateral)</td><td>16 (contralateral) and 16 (ipsilateral)</td><td>12 (contralateral) and 15 (ipsilateral)</td></tr>
<tr><td>Does Opening Side Relative to Side of Dominant Compression Affect Expansion of the Spinal Canal?</td><td>C3-4: 58 (contralateral) and 59 (ipsilateral)
C4-C5: 59 (contralateral) and 67 (ipsilateral)
C5-C6: 57 (contralateral) and 62 (ipsilateral)
C6-C7: 51 (contralateral) and 50 (ipsilateral)</td><td colspan="2">C3-4: 20 (contralateral) and 19 (ipsilateral)
C4-C5: 17 (contralateral) and 24 (ipsilateral)
C5-C6: 12 (contralateral) and 26 (ipsilateral)
C6-C7: 17 (contralateral) and 17 (ipsilateral)</td></tr>
</table>
